# Diet overlap and spatial segregation between two neotropical marsupials revealed by multiple analytical approaches

**DOI:** 10.1371/journal.pone.0181188

**Published:** 2017-07-12

**Authors:** Vanessa Villanova Kuhnen, Gustavo Quevedo Romero, Arício Xavier Linhares, Jeferson Vizentin-Bugoni, Erica Aline Correa Porto, Eleonore Zulnara Freire Setz

**Affiliations:** 1 Pós-Graduação em Ecologia, Instituto de Biologia, Universidade Estadual de Campinas (UNICAMP), Campinas, São Paulo, Brazil; 2 Departamento de Biologia Animal, Instituto de Biologia, Universidade Estadual de Campinas (UNICAMP), Campinas, São Paulo, Brazil; 3 Graduação em Ciências Biológicas, Instituto de Biologia, Universidade Estadual de Campinas (UNICAMP), Campinas, São Paulo, Brazil; University of Sydney, AUSTRALIA

## Abstract

Species co-existence depends on how organisms utilize their environment and resources. When two sympatric species are similar in some ecological requirements, their coexistence may arise from differences in resource use over time and/or space. Interactions among coexisting marsupials remain poorly understood, especially in the Neotropics. Here we combine spatial niche measurements, individual-resource networks, and isotopic niche approaches, to investigate the ecological strategies used by the Neotropical marsupials *Didelphis aurita* and *Metachirus nudicaudatus* to co-occur in an area of Serra do Mar State Park (southeast of Brazil). Both individual-resource networks and isotopic niche approaches indicate similar patterns of omnivory for both species. Isotopic analysis showed the species’ trophic niche to be similar, with 52% of overlap, and no differences between proportional contributions of each resource to their diets. Moreover, individual-resource network analysis found no evidence of diet nestedness or segregation. The trophic niche overlap observed was associated with spatial segregation between species. Despite using the same area over the year, *D*. *aurita* and *M*. *nudicaudatus* exhibited spatial segregation among seasons. These results illustrate that the detection of spatial segregation is scale-dependent and must be carefully considered. In conclusion, our findings provide a new perspective on the ecology of these two Neotropical marsupials by illustrating how the association of distinct but complementary methods can be applied to reach a more complete understanding of resource partitioning and species coexistence.

## Introduction

Species co-existence partly depends on how organisms utilize their resources and environment. All resources and conditions required by an organism to survive and reproduce are encompassed by the niche concept, which describes an *n*-dimensional hypervolume where dimensions represents distinct ecological requirement [1]. Hence, one way to analyze how species coexistence takes place is by measuring niche parameters and comparing them among species [2,3]. When two sympatric species have similar ecological requirements, there is niche overlap—which is the region of niche space shared by two or more species [4,5]. However, if niche dimensions of two species overlap completely, one species will probably supplant the other, leading to its local extinction (i.e. Gause’s competitive exclusion principle [6]). Therefore, coexistence may arise from niche shifts, for example, through partitioning resources along time and/or space [7,8].

The most tractable and frequently studied component of niche space is trophic niche width, often evaluated using dietary diversity [9]. Sympatric species can segregate trophic niches using different strategies, such as food type [10], foraging mode [11], spatial segregation [12], or circadian activity pattern [13]. A species’ diet can also respond to resource seasonality; thus, changes in food consumption across seasons can match fluctuations in food availability [14]. When resources are abundant, interspecific competition decreases and a higher trophic niche overlap is enabled [15]. However, diet overlap can also be observed when the demand for resources exceeds supply and species need to expand their trophic niche in order to make up for their nutritional demands [16]. A high degree of niche overlap may lead to competitive interactions [17, 18], leading to species displacement [19]. Therefore, other mechanisms of niche partitioning (e.g., spatial and/or time segregation) could allow co-existence among species with similar ecological requirements [20].

To investigate the structure of trophic relationships there are a variety of different approaches, such as individual-resource networks [21,22,23] and isotopic niche approaches [24,25]. Individual-resource networks are represented by two sets of nodes: one representing individuals of a population and one of food resource items or categories (*ca*. those found in fecal samples). The link between nodes represents the consumption of a given resource by an individual. Network structure can be investigated through metrics that characterize observed patterns of interactions [26]. There are some advantages associated with individual-resource networks based on fecal samples data, such as low costs and the possibility of collecting data from recaptured animals. Despite all the applicability, there are some inherent potential limitations associated to fecal samples data. For example, some items have different rates of digestion [27], thus, a sample may not contain traces from all items previously consumed. Besides that, the consumption of species without hard parts will probably not be tracked in the feces, biasing diet results. Food resource identification is not an accurate procedure as specific parts of the digested animals’ body are needed for reliable taxonomic identification. Those parts sometimes break down after digestion, leading to them being underrated in diet composition. Complementarily, some of these limitations can be remedied by using stable isotope signatures to provide a quantitative diet description [24,28].

Isotopic composition of animal tissues often contains a “signature” of the process that created it [29]. For example, stable carbon isotope ratios (δ^13^C) vary mostly according to the photosynthetic metabolism of plants and can be very effective tracers of different carbon sources [24]. The stable isotope commonly utilized to trace protein sources is nitrogen (δ^15^N). Therefore, the δ space (δ^13^C versus δ^15^N) is referred as the “isotopic niche” and has been comparable to the Hutchinson’s n-dimensional niche concept [1,29]. However, potential drawbacks in using this approach are: the unknown rate at which isotopic values from a given resource is assimilated by the tissues of the consumer, making it difficult to compare different tissues; the impossibility of using tissue samples for isotopic analyses from recaptured animals when bait is used; and, the challenge of accessing the isotopic signature of each consumed species. Therefore, using stable isotopes and fecal samples in conjunction provides a more complete representation of an individual or species’ dietary habit.

Here we combine spatial niche measurements, individual-resource networks analysis, and isotopic niche approaches to investigate the ecological strategies of two sympatric Neotropical marsupials (*Didelphis aurita* Wied-Neuwied, 1826 and *Metachirus nudicaudatus* (É. Geoffroy, 1803)) which co-occur in several Tropical Rainforest communities. Whereas *M*. *nudicaudatus* has a broad geographical range, *D*. *aurita* is restricted to Atlantic Rainforest biome [30]. *Didelphis aurita* is about three times larger than *M*. *nudicaudatus* [31], which makes the first a stronger competitor, as it is able to actively defend food resource [32,33]. Despite their morphological and behavioral differences, these two species are considered to be apparent competitors [34]. However, the processes underlying the coexistence of such species remain largely unknown.

As both, *D*. *aurita* and *M*. *nudicaudatus*, are mainly terrestrial, nocturnal, solitary, and feed on similar resources [35,36,37,38], strategies such as low trophic niche overlap or differential use of the space is expected, in order to promote their co-existence. Here, we investigated trophic niche overlap using both the isotopic approach [28] and analyzing the individual-resource network structure based on fecal samples. If, as suggested by literature [38], *D*. *aurita* is more generalist and *M*. *nudicaudatus* is predominantly insectivore, *M*. *nudicaudatus*’s diet would represent a subset of the broader diet of the generalist *D*. *aurita*. In this case, we would expect to find a nested structure for individual-resource network of both species [21,22] and overlapping isotopic niches. Alternatively, if individuals use distinct subsets of the available resources, we would expect a modular network structure, i.e. groups of individuals of the same species feeding on a subset of food items different than those exploited by the other species [39,22], with distinct isotopic niches.

## Materials and methods

### Study area

The study was conducted in Serra do Mar State Park, southeast Brazil (São Paulo State coast). Serra do Mar State Park comprises more than 300,000 ha of well-preserved rainforest—it is the largest protected Atlantic Rainforest remnant in Brazil [40]. The studied area is located 35 km northbound from the urban area of Ubatuba city, and 2 km from an isolated village (*Sertão da Fazenda*) (23°20’S and 44°50’W). The forest is characterized as lowland tropical evergreen, following Veloso et al. (1991) [41]. For a full description of the forest classification and structure, see Alves et al. (2010) [42] and Eisenlohr et al. (2013) [43]. The altitudes range from 43 to 89 m above sea level [43]. The regional climate is Tropical Humid, with hot summers and no dry season (Af/Cfa). Average annual precipitation is 2,500 mm, and monthly average temperature is 22°C [44].

### Collection and preparation of samples

From May 2013 to April 2015, trapping sessions of four consecutive nights were conducted every month. Tomahawk traps (45_×_16_×_16 cm) were used to capture animals, baited with banana, peanut butter and bacon. Traps were placed 20 m apart from each other, compounding a rectangular grid of 3.4 ha (fifteen lines with seven traps each, 280 m x 120 m). Such spacing represents the average distance used in previous studies [36,45], and allows to caught individuals to be sampled and released readly early in the morning, at the day after the trapping. This is particularly important as frequent rain and temperature changes may lead to termoregulatory imbalances and death of individuals trapped for long time. In addition, a Sherman trap (10 x 11 x 38 cm) fixed on tree branch, and a Tomahawk trap (45 x 16 x 16 cm) were fixed with L-bracket method (according to Graipel et al. (2003) [46]) were place at each field station during the first six months of study. However, both methods presented low capture success (only 4 individuals caught in 6 months); therefore, we settled the traps in the forest understory in order to reduce the time between each sample station, and to arrive more readily at stations with captured animals. [47]. Five sample stations were excluded because of hard access. Thus, we worked with one hundred traps total, summing up 9600 trapping nights of sampling effort. Traps were placed on a hardboard platform to minimize the contamination of feces with litter contents. After the second night of trapping, all baits were replaced with new ones. Individuals captured were marked in each ear with a 1-g monel ear tag (National Band and Tag Co.^®^), weighed, identified as to sex and age (according to Tyndale-Biscoe and MacKenzie (1976) [48]); the sample station was also recorded.

Over two-years of capture-mark-recapture, we trapped 68 individuals of *D*. *aurita* (334 captures) and 34 individuals of *M*. *nudicaudatus* (127 captures). Samples for diet analysis were collected along the first year of the study and only from adults. For isotopic analysis we sampled 11 males and 8 females of *D*. *aurita*, and 6 females and 7 males of *M*. *nudicaudatus*. For fecal analysis we sampled 9 females and 9 males of *D*. *aurita*, and 5 females and 10 males of *M*. *nudicaudatus*. The first time an individual was captured, we would cut a nail sample off each finger from the left-hind foot for analysis of stable isotope ratios of C (δ^13^C) and N (δ^15^N). Only the free margin (distal edge) of the nail was cutted. As there are no nerve endings at this part, anaesthesia was not needed. Nails are keratin based and the turnover of elements in such tissues are slower, therefore being considered metabolically inert tissues [49]. As stable isotopes are allocated into the keratin structure during growth, the dietary information for the period during which the tissue was synthesized is preserved [50]. However, the rate with which animals incorporate the isotopic values of the resources they consume can vary between individuals and tissue types [51]. Since these rates are still unknown for Neotropical marsupials, we decided to not collect and analyze nails from recaptured individuals, thus preventing bias from bait consumption. Given that *D*. *aurita* and *M*. *nudicaudatus* are close relatives (i.e. same subfamily [52], we assumed that their nails integrate diet over similar amounts of time. Under a stereoscope, nail samples were gently cleaned in a petri dish with water, then with alcohol 70%, and again with water. After cleansing, samples were oven dried at 60°C for 48 hours, and weighed up to ~1 mg into tin capsules for isotopic analyses.

We also collected samples of available diet sources for isotopic analysis, based on the literature about the species’ diet. Source samples were divided into six categories: small vertebrates (reptiles and rodents), fruits, and four different guilds of arthropods (herbivores, predators, omnivores and detritivores). Although we might miss some diet items that can be eventually consumed, those six categories encompass the main food resources used by *D*. *aurita* and *M*. *nudicaudatus* [35,36,37,45,53,54,55,56,57]. Fruits were collected opportunistically, and to collect small vertebrates and arthropods we used three lines with eight pitfalls each (pitfall volume of 60 liters). The source samples were oven dried at 60°C for 48 hours, and weighed up to ~1 mg into tin capsules for isotopic analyses.

Fecal samples were collected from the bottom of the traps where the animal spent the night. In order to avoid potential influences of baits on the detection and identification of the feeding items (e.g. via differential digestive efficiency after bait ingestion) and also keep comparable sampling effort among all individuals caught, we did not consider feces from recaptured individuals during the same trapping season. A total of 70 fecal samples were collected from 9 females and 9 males of *D*. *aurita*, and 5 females and 10 males of *M*. *nudicaudatus*. Samples were frozen until analyzed. After thawing, they were dissolved in water, and then filtered through a 1 mm mesh screen sieve under running tap water. The material retained on the sieve was dried at environment temperature and analyzed with a stereoscope. The taxonomic identification of prey items was validated by specialists.

The spatial use of this grid, for each species, was analyzed by the percentage of spatial overlap, calculated through Morisita’s Index of Overlap [2]. For this analysis, we considered all captures during the two years of the study (334 captures of 68 individuals of *D*. *aurita* and 127 captures of 34 individuals of *M*. *nudicaudatus*). In order to avoid any odoriferous interference of a species in the probability of forthcoming captures, cages were cleaned after each capture with alcohol 50%.

### Isotopic analyses

All isotopic analyses were conducted in the Stable Isotope Facility of the University of California, Davis, California, USA, using an online elemental analyzer (PDZ Europa ANCA-GSL, Sercon Ltd., Cheshire, UK) interfaced to a continuous-flow isotope ratio mass spectrometer (PDZ Europa 20–20).

For the statistical analyses, we used the convex hull (TA) approach to represent the overall dispersion within isotope niche space of *D*. *aurita* and *M*. *nudicaudatus* [25]. In order to analyze dietary similarity between the species, we compared the standard ellipse areas of the isotopic niches (SEA). SEA is estimated using Bayesian methods, which allow robust statistical comparisons between populations with unequal sample sizes, and contain the core mean of the population’s isotopic niche (40% of 10000 randomizations, [3]. Due to the small sample size, we employed a corrected sample size version of the SEA (SEAc, [3]). The overlap of SEAc was calculated as a measure of trophic niche overlap [28]. The relative contribution of each resource in *D*. *aurita* and *M*. *nudicaudatus* diets were estimated by the Bayesian mixing model SIAR [58].

Intra-population variation in diets was analyzed using two quantitative metrics originally developed by Layman et al. (2007) [25] and then adapted for a Bayesian inference by Jackson et al. [28]: a) *mean distance to centroid* (CD_b_) infers the population’s trophic diversity; b) *standard deviation of nearest neighbor distance* (SDNND_b_) infers the population’s trophic evenness. All metrics were calculated using the software package SIAR v4.2.2 [59] in R programming environment [60].

### Network analysis

We described the trophic interactions of *D*. *aurita* and *M*. *nudicaudatus* with their feeding items as a bipartite individual-resource network, consisting of two groups: captured individuals and the resources they consumed [22]. For such, we defined an incidence matrix where rows (i) correspond to individuals, and columns (j) correspond to each food resource found in fecal samples. For the network analysis, food resources were not divided into categories (as for isotopic analyses), but were considered all items found on feces, identified to the most accurate taxonomic level as possible. Each element (a_ij_) of the matrix corresponds to whether the consumption of a resource was recorded (a_ij_ = 1) or not (a_ij_ = 0). In order to account for potential biases caused by recaptures of the same individuals, we grouped into the same line food items consumed by each individual within a season. To evaluate the potential bias arisen from this choice, we also recalculated the network metrics using the raw data, i.e. without grouping recaptures, and found the results to be consistent regardless of the choice (S4 Table).

We searched for nestedness and modularity into this network to test whether individuals of a species exploit a subset of resources used by individuals of another (nested network structure) or whether individuals of both species exploit distinct subset of resources (modules coincident with species should be detected). We used the NODF metric, based on overlap and decreasing fill for both lines and columns of the matrix [61], and the modularity index (Q) with the optimization algorithm QuanBiMo, which searches for subsets of individual–resource in the network, yielding the largest degree of modularity [62]. Despite that *Q* was developed to explore quantitative networks (*see* [62]), it is also suitable for binary networks. To access metric significances, we compared the observed NODF and *Q* values to those obtained by 1,000 and 100 null model randomizations, respectively. The null model used was *shuffle*.*web*, which rearranges interactions randomly inside the matrix but keeps the dimensions of the observed matrix [63]. Due to the large computational time required by the algorithm QuanBiMo, we used less randomizations for modularity. Due to the stochastic optimization technique associated to this algorithm (*i*.*e*. simulated annealing), Q values can be slightly different among runs. Thus, for each network we chose the higher values from five independent runs set to 10^7^ swaps [63]. As the algorithm aims to find the highest modularity, getting the higher value among runs is a suitable procedure, as done by previous studies (e.g. [64]). The same was done for the null models in which we chose the higher Qs among five runs for each of the 100 randomizations. Metrics were considered significant when the observed value fell outside the 95% confidence interval expected by the null models (*i*.*e*. 1000 and 100 randomizations for NODF and *Q*, respectively). In order to evaluate temporal variation in the dietary structure, we split the network data into four seasons and repeated the analyses above to search for nestedness and modularity within a season. All analyses were run in R-package *bipartite* [65].

## Results

**Fig 1:**
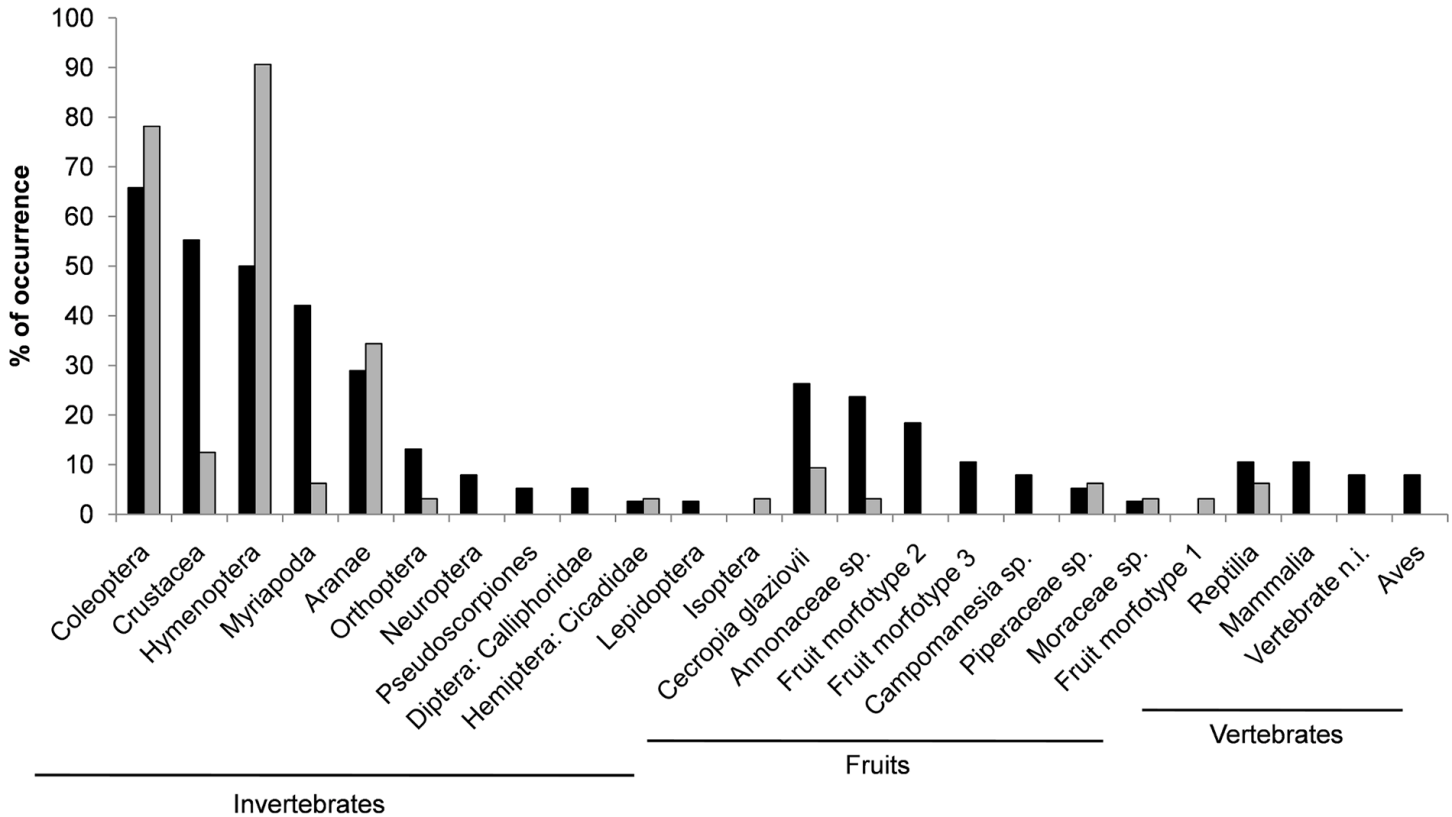
Occurrence of each food item (%) found in *Didelphis aurita* (black bars) and *Metachirus nudicaudatus* (grey bars) fecal samples.

**Fig 2:**
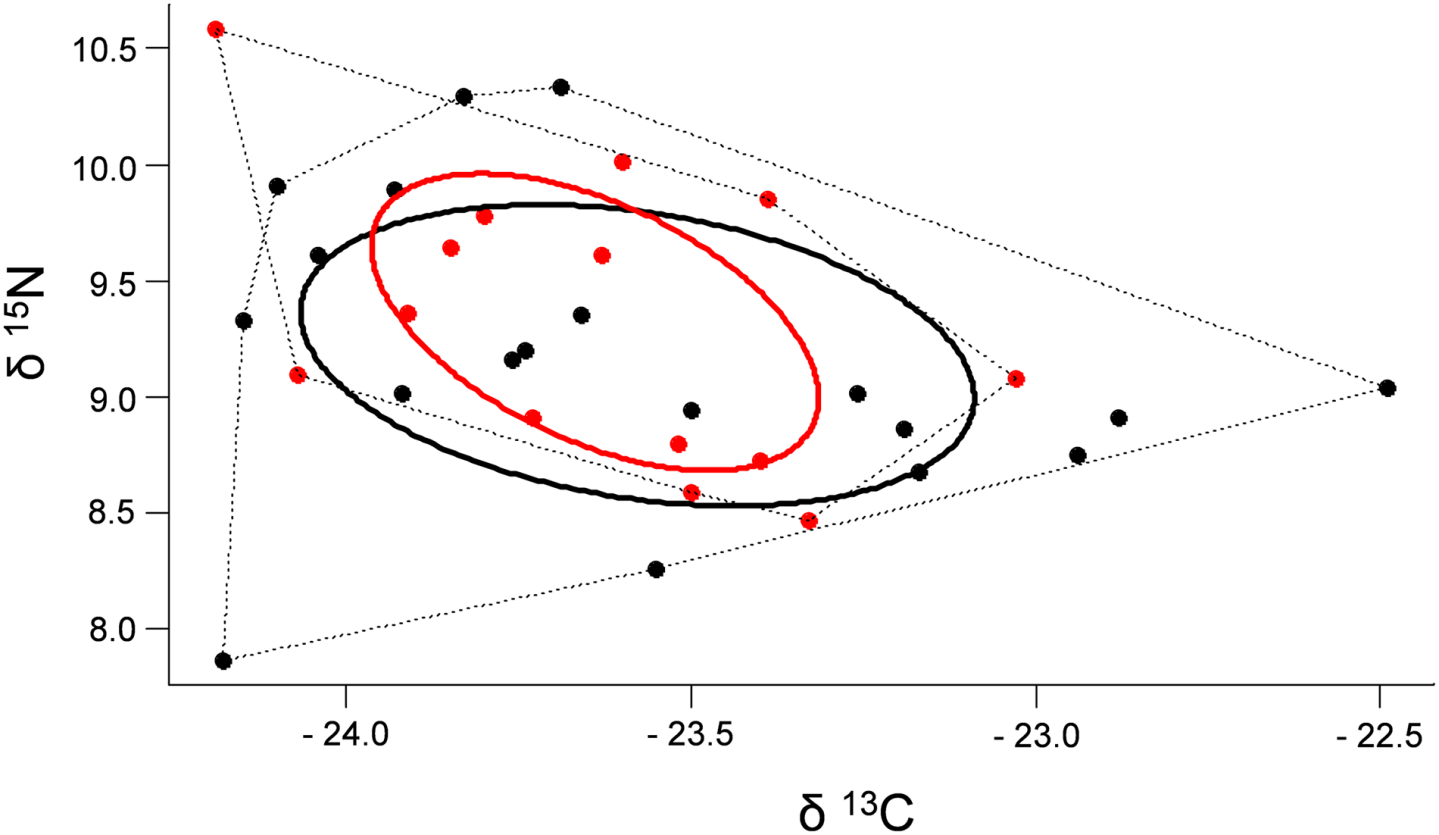
Stable isotope bi-plot illustrating the isotopic niche of *Didelphis aurita* (black) and *Metachirus nudicaudatus* (red). Each point represents an individual; solid lines represent the mean core of the population’s isotopic niche (SEAc), and; dotted lines represent the overall isotopic niche of the population (convex hull).

Arthropods were the main diet items found in scats of both mammal species (Fig 1; Supporting Information, S1 Table). Coleoptera and Crustacea were the items most frequently found in *D*. *aurita* scats and Hymenoptera and Coleoptera for *M*. *nudicaudatus*. Vertebrates were found more frequently in *D*. *aurita* scats than in *M*. *nudicaudatus*, which presented only reptile traces. A wider diversity of seeds was found in *D*. *aurita* scats and in a higher frequency than in *M*. *nudicaudatus*. Despite these differences, isotopic analyses showed that *D*. *aurita* and *M*. *nudicaudatus* feed in the same proportions for each food resource category. Mean values of δ^15^N for *D*. *aurita* and *M*. *nudicaudatus* were 9.18 (±0.10 SE) and 9.32 (±0.16 SE), respectively; and, mean values of δ^13^C were -23.58 (±0.10 SE) and -23.64 (±0.08 SE) for *D*. *aurita* and *M*. *nudicaudatus*, respectively. Standard ellipse areas for isotopic niches of *D*. *aurita* (SEAc = 0.95) and *M*. *nudicaudatus* (SEAc = 0.56) were similar (p = 0.34), and both species showed high trophic niche overlap (Fig 2). Population-level metrics for trophic structures were also similar for both species (Table 1).

**Table 1:** Population metrics of trophic structure for *Didelphis aurita* and *Metachirus nudicaudatus*.

	*Didelphis aurita*	*Metachirus nudicaudatus*
Sample size (n)	19	14
CDb	0.47	0.51
MNNDb	0.08	0.12
SDNNDb	0.12	0.14
SEAc	0.95	0.56
TA	2.28	1.27

CDb = mean distance to centroid; MNNDb = mean nearest neighbor distance; SDNNDb = standard deviation of MNNDb; SEAc = standard ellipse area; TA = total ellipse area

**Fig 3:**
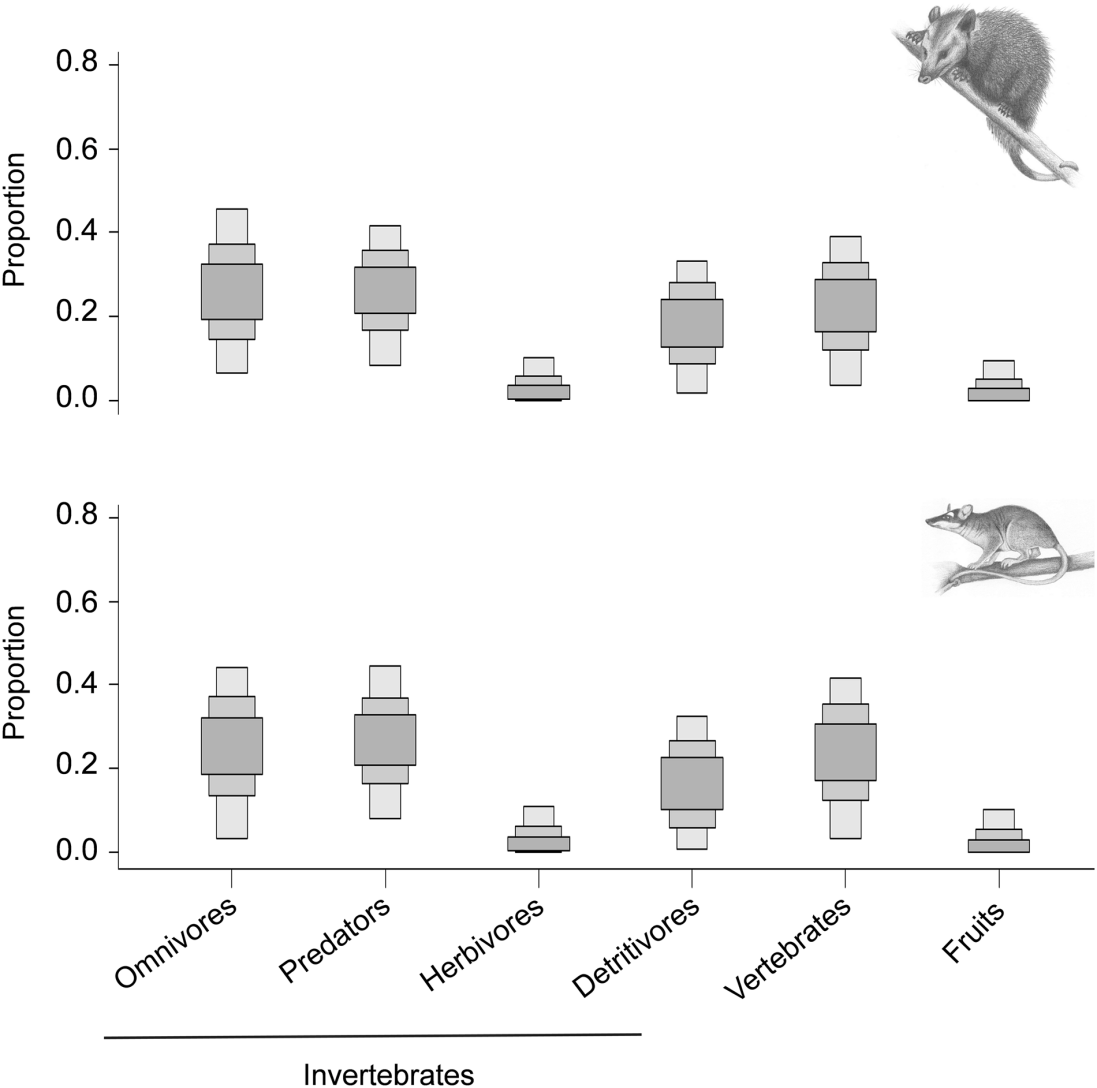
Contributions of potential food sources to the diets of *Didelphis aurita* (above) and *Metachirus nudicaudatus* (below). Boxes illustrate the relative proportions of each food source with 50%, 75% and 95% confidence intervals from dark to light grey.

SIAR dietary mixing model did not detect any significant differences between the proportion that each resource group contributed to *D*. *aurita* and *M*. *nudicaudatus* diets (isotopic values for all resource samples are shown in S2 Table). For both species, diets seem split into three main resource groups (Fig 3, S3 Table). Top food web invertebrates (omnivores and predators) were crucial resources (~52.5%) while vertebrates and detritivores showed a smaller but also important proportional contribution (~38%), with vertebrates contributing more (~21.5%) than detritivores (~0.16%). Sources from the base of the food web (fruits and herbivores) were less important dietary components, with a proportional contribution of 0.02%.

**Fig 4:**
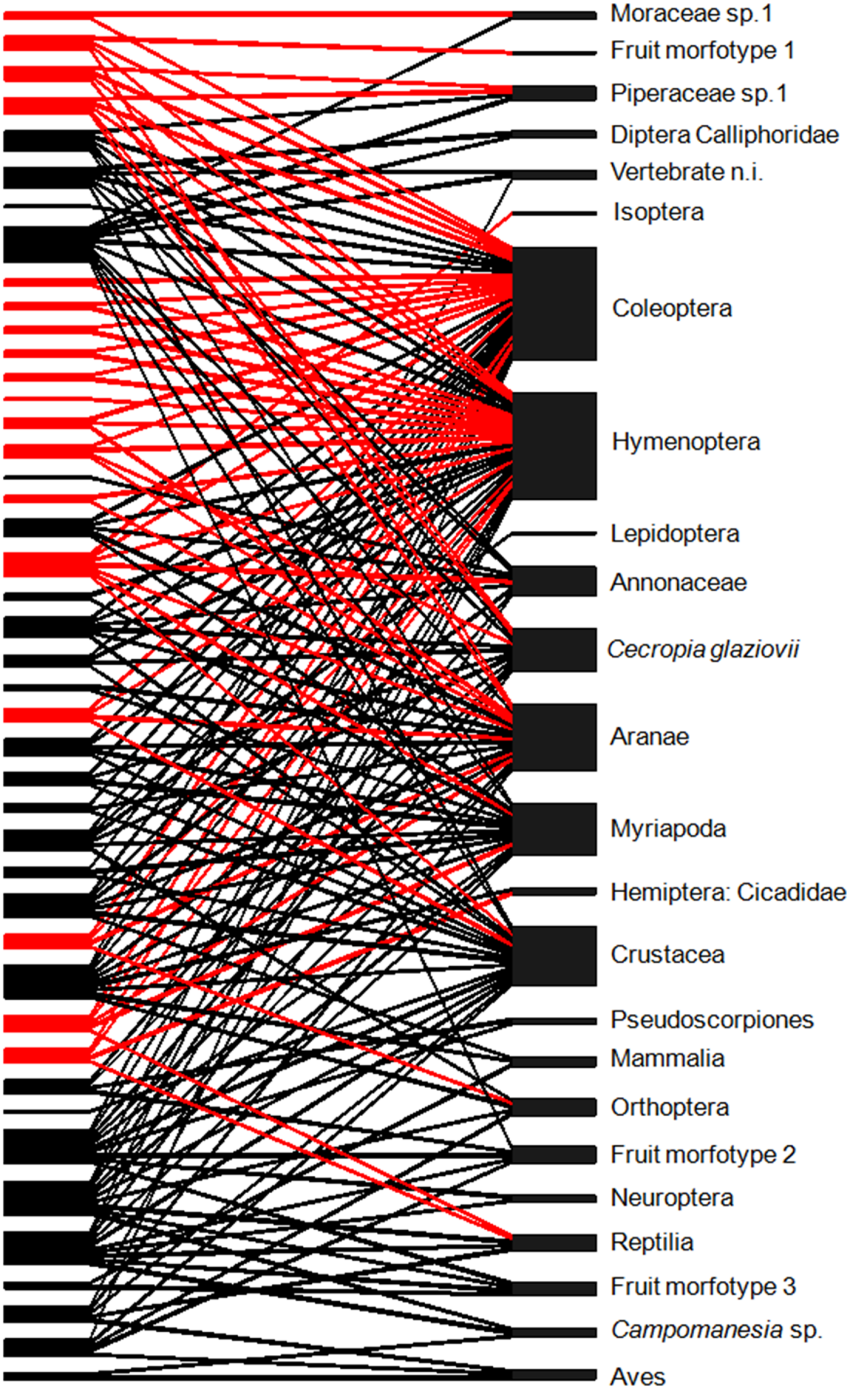
Individual-resource network for *Didelphis aurita* and *Metachirus nudicaudatus*. Links represent the consumption of a resource (right column) by each individual (left column). Black represents *D*. *aurita* and red *M*. *nudicaudatus individuals*.

We found no evidence of diet nestedness or segregation between both species (Fig 4), as there were no differences from observed and expected results by the null model, neither for the nestedness index (NODF = 51.09, 95% IC = 50.00–54.61) nor modularity (Q = 0.30, 95% IC = 0.28–0.30) for bipartite matrices. Moreover, their diet remained consistently unstructured over time as both nestedness and modularity did not differ from the results expected by the null models for the seasons (S4 Table).

When we merged 24 sampled months for spatial analysis, *D*. *aurita* and *M*. *nudicaudatus* overlapped 65% in the use of space. However, when thinner spatial scales were considered, *i*.*e*. seasons, spatial partitioning among the individuals became evident (Table 2). There was complete spatial segregation among individuals during the two springs and in the winter of 2013. Smaller spatial overlap was observed in the winter of 2014 and both autumns, staying between 14% and 24%. In the summer, individuals of both species had the highest grid use overlap (36%). There was no association between number of captures and spatial overlap. Higher capture rates for both species were recorded in the winter of 2013 and 2014, and there was no overlap of the grid area used (Table 2).

**Table 2:** Spatial niche overlap (%) between *Didelphis aurita* and *Metachirus nudicaudatus* in the Atlantic rain forest.

	2013	2014	2015
Summer	n.a.	36% (32/16)	0% (59/0)
Autumn	20% (23/19)	24% (26/26)	20% (33/12)
Winter	0% (53/18)	14% (56/23)	n.a.
Spring	0% (22/11)	0% (30/2)	n.a.

Percentages were calculated based on Morisita’s index. Values in parenthesis represent numbers of captures for each species (*D*. *aurita* / *M*. *nudicaudatus*). (n.a. represents season that were not sampled)

## Discussion

Through a combination of isotopic and network analyses, we showed that *D*. *aurita* and *M*. *nudicaudatus* present omnivore diets, contradicting previous literature based on fecal samples data [37,66,67]. Whereas *D*. *aurita* has been classified in the literature as the most generalist marsupial in the Atlantic Rainforest, *M*. *nudicaudatus* has been claimed omnivore but predominantly insectivore [37,66,67]. On the other hand, our findings suggest that *M*. *nudicaudatus* is in fact generalist, but more than initially thought of, which provides a new perspective to its ecological role in the community.

Our results support the idea that the use of stable isotopes in conjunction to fecal samples provides a more complete representation of a species’ dietary habits, and the importance of considering different arrays of analytical approaches for diet analyses [68]. Both approaches used to analyze the species’ diet indicate the same pattern of omnivory. Isotopic results also revealed that *D*. *aurita* and *M*. *nudicaudatus* diets encompass similar proportions of prey categories. On the other hand, fecal samples allowed a proper taxonomic identification of some dietary items [69]. It is worth highlighting that isotopic analysis demonstrated that herbivore invertebrates (*e*.*g*. Coleoptera) and fruits had a small contribution to *D*. *aurita* and *M*. *nudicaudatus* diets; whereas, in fecal samples, those were the categories most frequently registered. This is probably because Coleoptera’s elytra and fruit seeds are more difficult to digest. It is possible that the proportions of *D*. *aurita* and *M*. *nudicaudatus* diets change from area to area, however, our results evidence that these two sympatric marsupials have high trophic niche overlap and might compete for food resources.

Remarkably, both methodologies used here were not able to distinguish subtle differences between *D*. *aurita* and *M*. *nudicaudatus* diets, such as species consumed. Differences in species consumed might exist due to differences on their climbing ability. Specifically, *Didelphis aurita* is a good climber and can be found in all forest strata despite preferring the ground [36,70,71], whereas *M*. *nudicaudatus* has morphological adaptations to a cursorial behavior and is rarely captured on other forest strata than ground [72,73]. Therefore, it is possible that *D*. *aurita*’s climbing ability allows the species to consume different prey species from canopy. Besides, *M*. *nudicaudatus* has a longer molar series [74], which can increase the crushing, cutting and grinding surface, potentially decreasing the probability of finding resource parts in feces that allow proper identification. Thus, likewise for some other mammals, it is still a challenge to determine the array of species consumed by *M*. *nudicaudatus* and *D*. *aurita* [75,76,77,78].

In the studied area, *D*. *aurita* and *M*. *nudicaudatus* trophic niche overlap was associated to spatial segregation within seasons. The same pattern has also been described to *D*. *aurita* and *D*. *marsupialis* [32] and in other systems, such as for Australian rodents [79] and African grazers [20]. Our findings reveal that the temporal scale is crucial for the detection of spatial partitioning among species. Over the two years, *D*. *aurita* and *M*. *nudicaudatus* were captured in almost all areas of the grid, indicating that there was no difference in habitat use by both species. On the other hand, there was a dynamic spatial exclusion between species among seasons. This is expected as during seasons with more resource availability, such as a regular summer, interspecific competition for food resources decreases, leading to species potentially tolerating a higher spatial overlap; and the opposite might be expected for seasons with low resource availability [7], such as winter. Indeed, our data indicates that in summers there is a higher spatial overlap while winter has a lower one.

Overall, interactions among marsupials remain poorly understood, especially in the Neotropics. For other mammal species, such as otters [80] and canids [81], feces and urine are used as visual and olfactory signals. Cougars, for instance, leave scrapes along travel routes as intraspecific signs [82]. *Didelphis aurita* and *M*. *nudicaudatu*s have paracloacal scent glands that confer a peculiar smell to individuals [83,84] and are likely related to complex multifunctional behaviors [85,86]. The recognition of these olfactory signals in the environment could be a strategy of *M*. *nudicaudatus* to avoid encounters with *D*. *aurita*, that is three times bigger and very aggressive [87]. In this sense, further studies focusing on behavior may be valuable to better understand how interactions between these species result in the pattern of dietary overlap and space usage here described.

In conclusion, our findings provide evidence that both species are similarly omnivores but presenting preference for feeding on insects, and, despite using the same area over the year, exhibit spatial segregation among seasons. Therefore, spatial segregation is likely a major mechanism promoting coexistence by reducing competition for resources between these marsupials. Importantly, our results illustrate that the detection of spatial segregation is scale dependent and might be considered carefully. Finally, this paper provides a new perspective on the ecology of these two Neotropical marsupials, and also illustrates how the association of distinct but complementary methods can be applied to reach a more complete understanding of resource partitioning and species coexistence.

## Supporting information

S1 Table Frequencies of occurrence (%) of food items found in *Didelphis aurita* and *Metachirus nudicaudatus* fecal samples. This is the S1 Table legend.
(PDF)

S2 Table δ13C and δ15N values for putative resources to the diet of *Didelphis aurita* and *Metachirus nudicaudatus*. This is the S2 Table legend.
(PDF)

S3 Table Relative contribution of putative resources to the diet of *Didelphis aurita* and *Metachirus nudicaudatus*. This is the S3 Table legend.
(PDF)

S4 Table Nestedness (NODF) and Modularity (Q) for *Didelphis aurita* and *Metachirus nudicaudatus* individual-resource networks. This is the S4 Table legend.
(PDF)

## References

[1] Hutchinson GE. Concluding remarks. Cold Spring Harb. Sym. 1957;22: 415–427.

[2] Krebs CJ. Ecological methodology. 2nd ed Boston: Addison Wesley Longman; 1999.

[3] Jackson AL, Inger R, Parnell AC, Bearhop S. Comparing isotopic niche widths among and within communities: SIBER–Stable Isotope Bayesian Ellipses in R. J. Anim. Ecol. 2011;80: 595–602. doi: 10.1111/j.1365-2656.2011.01806.x 21401589 10.1111/j.1365-2656.2011.01806.x

[4] Colwell RK, Futuyma DJ. On the measurement of niche breadth and overlap. Ecology. 1971;52: 567–576. 10.2307/1934144 28973805

[5] Svendsen GM, Romero MA, Williams GN, Gagliardini DA, Crespo EA, Dans SL, et al Environmental niche overlap between common and dusky dolphins in north Patagonia, Argentina. PLoS One. 2015;10: e0126182 doi: 10.1371/journal.pone.0126182 26091542 10.1371/journal.pone.0126182 PMC4474694

[6] Gause GF. The struggle for existence. Baltimore: Williams and Wilkins; 1934

[7] Schoener TW. Resource partitioning in ecological communities. Science. 1974;185: 27–39. doi: 10.1126/science.185.4145.27 17779277 10.1126/science.185.4145.27

[8] Benadi G. Requirements for plant coexistence through pollination niche partitioning. P. Roy. Soc. B. 2015;282: 20150117. 10.1098/rspb.2015.0117 PMC4590466 26108627

[9] Bearhop S, Adams CE, Waldron S, Fuller RA, MacLeod H. Determining trophic niche width: a novel approach using stable isotope analysis. J. Anim. Ecol. 2004;73: 1007–1012.

[10] Mohd-Azlan J, Noske RA, Lawes MJ. Resource partitioning by mangrove bird communities in North Australia. Biotropica. 2014;46: 331–340.

[11] Nakano S, Fausch KD, Kitano S. Flexible niche partitioning via a foraging mode shift: a proposed mechanism for coexistence in stream-dwelling charrs. J. Anim. Ecol. 1999;68: 1079–1092.

[12] Browning NE, Cockcroft VG, Worthy GAJ. Resource partitioning among South African delphinids. J. Exp. Mar. Biol. Ecol. 2014;457: 15–21.

[13] Kronfeld-Schor N, Dayan T. The dietary basis for temporal partitioning: food habits of coexisting Acomys species. Oecologia. 1999;121: 123–128. doi: 10.1007/s004420050913 28307881 10.1007/s004420050913

[14] Correa SB, Winemiller KO. Niche partitioning among frugivorous fishes in response to fluctuating resources in the Amazonian floodplain forest. Ecology. 2014;95: 210–224. 24649660 10.1890/13-0393.1

[15] Reid REB, Greenwald EN, Wang Y, Wilmers CC. Dietary niche partitioning by sympatric *Peromyscus boylii* and *P*. *californicus* in a mixed evergreen forest. J. Mammal. 2013;94: 1248–1257.

[16] Baldi R, Pelliza-Sbriller A, Elston D, Albon S. High potential for competition between guanacos and sheep in Patagonia. J. Wildlife Manage. 2004;68: 924–938.

[17] Righetti J, Fox BJ, Croft DB. Behavioural mechanisms of competition in small dasyurid marsupials. Aust. J. Zool. 2000;48: 561–576.

[18] Harris DB, McDonald DW. Interference competition between introduced black rats and endemic Galápagos rice rats. Ecology. 2007;88: 2330–2344. 17918410 10.1890/06-1701.1

[19] Remonti L, Balestrieri A, Ruiz-González A, Gómez-Moliner BJ, Capelli E, Prigioni C. Intraguild dietary overlap and its possible relationship to the coexistence of mesocarnivores in intensive agricultural habitats. Popul. Ecol. 2012;54: 521–532.

[20] Macandza VA, Owen-Smith N, Cain JW. Dynamic spatial partitioning and coexistence among tall grass grazers in an African savanna. Oikos. 2012;121: 891–898.

[21] Araújo MS, Martins EG, Cruz LD, Fernandes FR, LInhares AX, Reis SF, et al Nested diets: a novel pattern of individual-level resource use. Oikos 2010;119: 81–88.

[22] Pires MM, Guimarães PR Jr, Araújo MS, Giaretta AA, Costa JCL, Reis SF. The nested assembly of individual-resource networks. J. Anim. Ecol. 2011;80: 896–903. doi: 10.1111/j.1365-2656.2011.01818.x 21644976 10.1111/j.1365-2656.2011.01818.x

[23] Cantor M, Pires MM, Longo GO, Guimarães PR Jr., Setz EZF. Individual variation in resource use by opossums leading to nested fruit consumption. Oikos. 2013;122: 1085–1093.

[24] Crawford K, McDonald RA, Bearhop S. Applications of stable isotope techniques to the ecology of mammals. Mammal Rev. 2008;38: 87–107.

[25] Layman CA, Arrington DA, Montaña CG, Post DM. Can stable isotope ratios provide for community-wide measures of trophic structure? Ecology. 2007;88: 42–48. 17489452 10.1890/0012-9658(2007)88[42:csirpf]2.0.co;2

[26] Costa LDF, Rodrigues CF, Travieso G, Villas Boas PR. Characterization of complex networks: a survey of measurements. Adv. Phys. 2007;56: 167–242.

[27] Putman RJ. Facts from feces. Mammal Rev. 1984;14: 79–97.

[28] Jackson MC, Donohue I, Jackson AL, Britton JR, Harper DM, Grey J. Population-level metrics of trophic structure based on stable isotopes and their application to invasion ecology. PLoS One. 2012;7: e31757 doi: 10.1371/journal.pone.0031757 22363724 10.1371/journal.pone.0031757 PMC3283663

[29] Newsome SD, del Rio CM, Bearhop S, Phillips DL. A niche for isotopic ecology. Front. Ecol. Environ. 2007;5: 429–436.

[30] Melo GL, Sponchiado J. Distribuição geográfica dos marsupiais no Brasil In: Cáceres NC, editor. Os marsupiais do Brasil: biologia, ecologia e conservação. Campo Grande: Editora UFMS; 2012 pp. 385–406.

[31] Rossi RV, Brandão MV, Carmignotto AP, Miranda CL, Cherem JJ. In: Cáceres NC, editor. Os marsupiais do Brasil: biologia, ecologia e conservação. Campo Grande: Editora UFMS; 2012 pp. 385–406.

[32] Cáceres NC, Machado AF. Spatial, dietary and temporal niche dimensions in ecological segregation of two sympatric, congeneric marsupial species. Open Ecol. J. 2013;6: 10–23.

[33] Breviglieri CPB, Kuhnen VV. Resource-defense behaviour: first report of an agonistic interaction between the opossum *Didelphis aurita* and the bat *Artibeus lituratus*. Folia Zool. 2016;65: 243–247.

[34] Kuhnen VV, Aguiar MAM, Gonçalves AZ, Setz EZF. Realized trophic niche driven by apparent competition: an example with marsupials. Biotropica;2017, forthcoming.

[35] Santori RT, Moraes DA, Cerqueira R. Diet composition of *Metachirus nudicaudatu*s and *Didelphis aurita* (Marsupialia, Didelphoidea) in Southeastern Brazil. Mammalia. 1995;59: 511–516.

[36] Grelle CEV. Forest structure and vertical stratification of small mammals in a secondary Atlantic Forest, Southeastern Brazil. Stud. Neotrop. Fauna E. 2003;38: 81–85.

[37] Cáceres NC. Diet of three didelphid marsupials (Mammalia, Didelphimorphia) in southern Brazil. Mamm. Biol. 2004;69: 430–433.

[38] Santori RT, Lessa LG, Astúa DM. Alimentação, nutrição e adaptações alimentares de marsupiais brasileiros In: Cáceres NC, editor. Os marsupiais do Brasil: biologia, ecologia e conservação. Campo Grande: Editora UFMS; 2012 pp. 385–406.

[39] Araújo MS, Guimarães PR Jr, Svanbãck R, Pinheiro A, Guimarães P, Reis SF, et al Network analysis reveals contrasting effects of intraspecific competition on individual vs. population diets. Ecology. 2008;89: 1981–1993. 18705384 10.1890/07-0630.1

[40] Ribeiro MC, Metzger JP, Martensen AC, Ponzoni FJ, Hirota MM. The Brazilian Atlantic Forest: how much is left, and how is the remaining forest distributed? Implications for conservation. Biol. Conserv. 2009;142: 1141–1153.

[41] Veloso HP, Lima JCA, Rangel Filho ALR. Classificação da vegetação brasileira adaptada a um sistema universal. Rio de Janeiro: IBGE; 1991.

[42] Alves LF, Vieira AS, Scaranello MA, Camargo PB, Santos FAM, Joly CA, et al Forest structure and live aboveground biomass variation along an elevational gradient of tropical Atlantic moist forest (Brazil). Forest Ecol. Manage. 2010;260: 679–691.

[43] Eisenlohr PV, Alves LF, Bernacci LC, Padgurschi MCG, Torres RB, Prata EMB, et al Disturbances, elevation, topography and spatial proximity drive vegetation patterns along an altitudinal gradient of a top biodiversity hotspot. Biodivers. Conserv. 2013;22: 2767–2783.

[44] CIIAGRO (Centro Integrado de Informações Agrometeorológicas) 2015. CIIAGRO Data Base - http://www.ciiagro.sp.gov.br/ciiagroonline/MenuMonClim.htm.

[45] Freitas SR, Moraes DA, Santori RT, Cerqueira R. Habitat preference and food use by *Metachirus nudicaudatus* and *Didelphis aurita*. Braz. J. Biol. 1997;57: 93–98.

[46] Graipel ME, Cherem JJ, Miller PRM, Glock L. Trapping small mammals in the forest understory: a comparison of three methods. Mammalia. 2003;67: 551–558.

[47] Kuhnen VV, Setz EZF. Bem-estar de pequenos mamíferos capturados em armadilhas de grade. Bol.Soc.Bras.Mastozool. 2016;75: 1–7.

[48] Tyndale-Biscoe CH, MacKenzie RB. Reproduction in *Didelphis marsupialis* and *D*. *albiventris* in Colombia. J. Mammal. 1976;57: 249–265. 932567

[49] Rubenstein DR, Hobson KA. From birds to butterflies: animal movement patterns and stable isotopes. Trends Ecol. Evol. 2004;19: 256–263. doi: 10.1016/j.tree.2004.03.017 16701265 10.1016/j.tree.2004.03.017

[50] Hobson KA. Tracing origins and migration of wildlife using stable isotopes: a review. Oecologia. 1999;120: 314–326. doi: 10.1007/s004420050865 28308009 10.1007/s004420050865

[51] Caut S, Angulo E, Courchamp F. Variation in discrimination factors (δ^15^N and δ^13^C): the effect of diet isotopic values and applications for diet reconstruction. J. Appl. Ecol. 2009;46: 443–453.

[52] Jansa SA, Barker FK, Voss RS. The early diversification history of Didelphid marsupials: a window into South America’s “splendid isolation”. Evolution. 2014;68: 684–695. doi: 10.1111/evo.12290 24125654 10.1111/evo.12290

[53] Carvalho FMV, Pinheiro PS, Fernandez FAS, Nessimian JL. Diet of small mammals in Atlantic Forest fragments in Southeastern Brazil. Revista Brasileira de Zoociências. 1999;1: 91–101.

[54] Cáceres NC, Monteiro-Filho ELA. Food habits, home range and activity of *Didelphis aurita* (Mammalia, Marsupialia) in a forest fragment of Southern Brazil. Stud. Neotrop. Fauna Environ. 2001;36: 85–92.

[55] Ceotto P, Finotti R, Santori R, Cerqueira R. Diet variation of the marsupials *Didelphis aurita* and *Philander frenatus* (Didelphimorphia, Didelphidae) in a rural area of Rio de Janeiro state, Brazil. Mastozoologia Neotropical. 2009;16: 49–58.

[56] Lessa LG, Costa FN. Diet and seed dispersal by five marsupials (Didelphimorphia: Didelphidae) in a Brazilian cerrado reserve. Mamm. Biol. 2010;75: 10–16.

[57] Lessa LG, Geise L. Food habits of *Metachirus nudicaudatus* (Didelphimorphia, Didelphidae) in a Brazilian Cerrado: diet composition and dietary seasonality. Stud. Neotrop. Fauna E. 2014;49: 75–78.

[58] Parnell AC, Inger R, Bearhop S, Jackson AL. Source partitioning using stable isotopes: coping with too much variation. PLoS One. 2010;5: e9672 doi: 10.1371/journal.pone.0009672 20300637 10.1371/journal.pone.0009672 PMC2837382

[59] Parnell A, Jackson A. Siar: Stable Isotope Analysis in R. R package version 4.2.2. 2013.

[60] R Core Team. R: A language and environment for statistical computing. https://www.R-project.org/. Accessed 10 October 2016.

[61] Almeida-Neto M, Guimarães PR, Guimarães PR Jr, Loyola RD, Ulrich W. A consistent metric for nestedness analysis in ecological systems: reconciling concept and measurement. Oikos. 2008;117: 1227–1239.

[62] Dormann CF, Strauss R. A method for detecting modules in quantitative bipartite networks. Method. Ecol. Evol. 2014;5: 90–98.

[63] Dormann CF, Fründ J, Blüthgen N, Gruber B. Indices, graphs and null models: analyzing bipartite ecological networks. Open Ecol. J. 2009;2: 7–24.

[64] González AMM, Dalsgaard B, Nogués-Bravo D, Graham CH, Schleuning M, Maruyama PK. The macroecology of phylogenetically structured hummingbird–plant networks. Global Ecol. Biogeogr. 2015;24: 1212–1224.

[65] Dormann CF, Gruber B, Fründ J. Introducing the bipartite package: analysing ecological networks. R news. 2008;8: 8–11.

[66] Vieira EM, Astúa D. Carnivory and insectivory in Neotropical marsupials In: Jones M., C. Dickman C, Archers M, editors. Predators with pouches: the biology of carnivorous marsupials. CSIRO Publishing; 2006 pp. 271–284.

[67] Paglia AP, Fonseca GAB, Rylands AB, Herrmann G, Aguiar LMS, Chiarello AG. Annotated Checklist of Brazilian Mammals. 2nd ed. Occasional Papers In Conservation Biology. 2012;6:1–76.

[68] Burns JM, Trumble SJ, Castellini MA, Testa JW. The diet of Weddell seals in McMurdo Sound, Antarctica as determined from scat collections and stable isotope analysis. Polar Biology. 1998;19: 272–282.

[69] Dickman CR, Huang C. The Reliability of fecal analysis as a method for determining the diet of insectivorous mammals. J. Mammal. 1988;69: 108–113.

[70] Passamani M. Análise da comunidade de marsupiais em Mata Atlântica de Santa Teresa, Espírito Santo. Bol. Mus. Bio. Mello Leitão. 2000;11/12: 215–228.

[71] Cunha AA, Vieira MV. Support diameter, incline, and vertical movements of four didelphid marsupials in the Atlantic forest of Brazil. J. Zool. 2002;258: 419–426.

[72] Argot C. Functional-adaptive analysis of the hindlimb anatomy of extant marsupials and the paleobiology of the Paleocene marsupials *Mayulestes ferox* and *Pucadelphys andinus*. J. Morphol. 2002;253: 76–108. doi: 10.1002/jmor.1114 11981806 10.1002/jmor.1114

[73] Astúa D, Moura RT, Grelle CEV, Fonseca MT. Influence of baits, trap type and position for small mammal capture in a Brazilian lowland Atlantic Forest. Biol. Mus. Biol. Leitão.2006;19: 31–44.

[74] Astúa D, Hingst-Zaher E, Marcus L, Cerqueira R. A geometric morphometric analysis of cranial and mandibular shape variation of *Didelphid marsupials*. Hystris. 2000;11: 115–130.

[75] Bowen WD, Iverson SJ. Methods of estimating marine mammal diets: A review of validation experiments and sources of bias and uncertainty. Mar. Mamm. Sci. 2012;29: 719–754.

[76] Deagle BE, Chiaradia A, McInnes J, Jarman SN. Pyrosequencing faecal DNA to determine diet of little penguins: is what goes in what comes out? Conserv Genet. 2010;11: 2039.

[77] Klare U, Kamler JF, MacDonald DW. A comparison and critique of different scat-analysis methods for determining carnivore diet. Mammal Review. 2011;41: 294–312.

[78] Pompanon F, Deagle BE, Symondson WO, Brown DS, Jarman SN, Taberlet P. Who is eating what: diet assessment using next generation sequencing. Mol Ecol. 2012;21:1931–50. doi: 10.1111/j.1365-294X.2011.05403.x 22171763 10.1111/j.1365-294X.2011.05403.x

[79] Maitz WE, Dickman CR. Competition and habitat use in native Australian *Rattus*: is competition intense, or important? Oecologia. 2001;128: 526–538. doi: 10.1007/s004420100689 28547398 10.1007/s004420100689

[80] McDonald SM, Mason CF. Observations on the marking behavior of a costal population of otters. Acta Theriol. 1980;25: 245–253.

[81] Dietz JM. Ecology and social organization of the maned wolf (Chrysocyon brachyurus).—Smithson. Contr. Zool. 1984;392: 1–51.

[82] Logan KA, Sweanor LL. Behavior and social organization of a solitary carnivore In: Hornocker M, Negri S, editors. Cougar Ecology and Conservation. Chicago: The Univ. of Chicago Press; 2010 Pp. 105–117.

[83] Helder-José H, Freymuller E. A morphological and ultra structural study of the paracloacal (scent) glands of the marsupial *Metachirus nudicaudatus* Geoffroy, 1803. Acta Anat. 1995;153: 31–38. 8560957

[84] Munhoz COG, Merzel J. Morphology and histochemistry study on perianal glands of the opossum (*Didelphis aurita*). Acta Anat. 1967;68: 258–271. 5587520

[85] Thomson JA, Pears FN. The functions of the anal glands of the brushtail possum. Victorian Naturalist. 1962;78: 306–308.

[86] Helder-José H. Glândulas paracloacais de marsupiais In: Cáceres NC, editor. Os marsupiais do Brasil: Biologia, Ecologia e Conservação. Campo Grande: Editora UFMS; 2012 pp. 185–196.

[87] Moura MC, Vieira MV, Cerqueira R. Occasional intraguild predation structuring small mammal assemblages: the marsupial *Didelphis aurita* in the Atlantic Forest of Brazil. Austral Ecol. 2009;34: 481–489.

