# Supplementary material for: Diet overlap and spatial segregation between two neotropical marsupials revealed by multiple analytical approaches
Source: PLoS One. 2017 Jul 12;12(7):e0181188. doi: 10.1371/journal.pone.0181188 (PMC5507539; doi:10.1371/journal.pone.0181188)
Supplement: S1 Table — This is the S1 Table legend. (PDF) [file pone.0181188.s001.pdf]

Table S1. Frequencies of occurrence (in %) of food items found in *Didelphis aurita* and M*etachirus nudicaudatus* fecal samples.

	Didelphis aurita (n=38)		***Metachirus nudicaudatus*** (n=32)	
**Arthropods**				
Coleoptera	25	(65.8)	25	(78.1)
Crustacea	21	(55.3)	4	(12.5)
Hymenoptera	19	(50.0)	29	(90.6)
Myriapoda	16	(42.1)	2	(6.3)
Aranae	11	(28.9)	11	(34.4)
Orthoptera	5	(13.2)	1	(3.1)
Neuroptera	3	(7.9)	0	(0.0)
Diptera: Calliphoridae	2	(5.3)	0	(0.0)
Pseudoscorpiones	2	(5.3)	0	(0.0)
Hemiptera: Cicadidae	1	(2.6)	1	(3.1)
Lepidoptera	1	(2.6)	0	(0.0)
Isoptera	0	(0.0)	1	(3.1)
**Vertebrates**				
Reptilia	4	(10.5)	2	(6.3)
Mammalia	4	(10.5)	0	(0.0)
Aves	3	(7.9)	0	(0.0)
Vertebrate n.i.	3	(7.9)	0	(0.0)
**Fruits**				
Cecropia glaziovii	10	(26.3)	3	(9.4)
Annonaceae sp.1	9	(23.7)	1	(3.1)
Campomanesia sp.	3	(7.9)	0	(0.0)
Piperaceae sp.1	2	(5.3)	2	(6.3)
Moraceae sp.1	1	(2.6)	1	(3.1)
Morfotype 1	0	(0.0)	1	(3.1)
Morfotype 2	7	(18.4)	0	(0.0)
Morfotype 3	4	(10.5)	0	(0.0)

(n.i. = not identified)
