# Supplementary material for: Diet overlap and spatial segregation between two neotropical marsupials revealed by multiple analytical approaches
Source: PLoS One. 2017 Jul 12;12(7):e0181188. doi: 10.1371/journal.pone.0181188 (PMC5507539; doi:10.1371/journal.pone.0181188)
Supplement: S2 Table — This is the S2 Table legend. (PDF) [file pone.0181188.s002.pdf]

Table S2. δ^{13}C and δ^{15}N values for putative resources to the diet of *Didelphis aurita* and *Metachirus nudicaudatus*.

Guild	Class	Order	Family / Sp.		δ15N	δ13C
**Omnivores**						
	Arachnida	Opiliones	-		7.71	-25.83
	Insecta	Orthoptera	Anostostomatidae		5.31	-27.14
	Insecta	Hymenoptera	Formicidae		4.81	-26.76
	Insecta	Hymenoptera	Formicidae		7.68	-26.87
				Mean	6.37	-26.65
				SD	± 1.53	± 0.57
**Predators**						
	Arachnida	Araneae	Lycosidae		7.38	-27.57
	Arachnida	Araneae	Lycosidae		8.25	-26.87
	Arachnida	Araneae	Lycosidae		5.07	-27.66
	Arachnida	Araneae	Theraphosidae		8.58	-26.83
	Arachnida	Scorpiones	-		8.57	-26.69
	Chilopoda	-	-		7.40	-27.91
	Insecta	Hymenoptera	Pompilidae		9.40	-26.84
	Insecta	Hymenoptera	Pompilidae		7.48	-27.03
	Insecta	Hymenoptera	Pompilidae		12.83	-25.43
	Insecta	Hymenoptera	Ponerinae		9.14	-26.24
	Insecta	Hymenoptera	Vespidae		6.65	-27.68
				Mean	8.25	-26.98
				SD	± 1.95	± 0.72
**Herbivores**						
	Insecta	Coleoptera	Cerambycidae		-1.30	-25.94
	Insecta	Coleoptera	Cerambycidae		-1.45	-26.24
	Insecta	Orthoptera	Tettigoniidae		5.28	-27.78
	Insecta	Orthoptera	Tettigoniidae		4.22	-27.97
	Insecta	Phasmatodea	-		0.37	-33.03
	Insecta	Phasmatodea	-		-0.22	-33.16
				Mean	1.15	-29.02
				SD	± 2.89	± 3.25
**Detritivores**						
	Diplopoda	-	-		6.78	-23.17
	Diplopoda	-	-		2.63	-26.22
	Insecta	Blattodea	-		3.16	-27.38
	Insecta	Blattodea	-		4.42	-26.37
	Insecta	Coleoptera	-		4.52	-28.20
	Insecta	Coleoptera	Passalidae		0.70	-27.36
	Insecta	Coleoptera	Scarabaeidae		4.88	-26.49
	Insecta	Coleoptera	Staphilinidae		6.93	-25.52
				Mean	4.25	-26.34
				SD	± 2.08	± 1.53
**Vertebrates**						

	Mammalia	Rodentia	*Euryoryzomys russatus*	6.33	-26.09
	Mammalia	Rodentia	*Euryoryzomys russatus*	6.48	-26.05
	Mammalia	Rodentia	*Juliomys pictipes*	4.76	-27.79
	Mammalia	Rodentia	*Oligoryzomys nigripes*	4.95	-31.30
	Mammalia	Rodentia	*Oxymycterus* sp.	10.26	-25.32
	Reptilia	Squamata	-	8.93	-23.61
			Mean	6.95	-26.69
			SD	± 2.20	± 2.62
**Fruits**					
			Morfotype 1	-0.06	-31.42
			Morfotype 2	1.15	-26.79
			Morfotype 3	5.78	-29.91
			Morfotype 4	4.74	-35.78
			Morfotype 5	-1.75	-28.10
			Morfotype 6	0.78	-29.59
			Morfotype 7	2.46	-28.02
			Morfotype 8	3.05	-33.11
			Morfotype 9	-1.09	-27.94
			Morfotype 10	1.16	-35.03
			Morfotype 11	0.42	-29.91
			Morfotype 12	0.05	-29.99
			Morfotype 13	2.80	-31.44
			Morfotype 14	1.37	-28.17
			Morfotype 15	2.21	-28.45
			Morfotype 16	0.02	-28.90
			Morfotype 17	0.21	-28.98
			Morfotype 18	2.01	-29.89
			Morfotype 19	-1.77	-29.84
			Morfotype 20	-2.11	-27.54
			Morfotype 21	-0.42	-29.23
			Morfotype 22	3.20	-25.85
			Morfotype 23	-2.36	-30.58
			Morfotype 24	0.95	-29.16
			Morfotype 25	3.26	-35.59
			Mean	1.04	-29.97
			SD	± 2.09	± 2.57
