# Supplementary material for: Diet overlap and spatial segregation between two neotropical marsupials revealed by multiple analytical approaches
Source: PLoS One. 2017 Jul 12;12(7):e0181188. doi: 10.1371/journal.pone.0181188 (PMC5507539; doi:10.1371/journal.pone.0181188)
Supplement: S3 Table — This is the S3 Table legend. (PDF) [file pone.0181188.s003.pdf]

Table S3. Relative contribution of putative resources to the diet of *Didelphis aurita* and *Metachirus nudicaudatus*.

Species	Source category	Confidence intervals		Mode	Mean
		Low 95%	High 95%		
Didelphis aurita					
	Omnivores	0.07	0.46	0.27	0.26
	Predators	0.08	0.41	0.26	0.26
	Herbivores	0.00	0.10	0.01	0.04
	Detritivores	0.02	0.33	0.19	0.18
	Vertebrates	0.04	0.39	0.21	0.22
	Fruits	0.00	0.09	0.01	0.03
	Nitrogen SD	0.00	0.77	0.07	0.30
	Carbon SD	1.41	3.66	2.26	2.47
Metachirus nudicaudatus					
	Omnivores	0.04	0.44	0.26	0.25
	Predators	0.08	0.44	0.26	0.27
	Herbivores	0.00	0.11	0.01	0.04
	Detritivores	0.01	0.33	0.14	0.17
	Vertebrates	0.03	0.41	0.22	0.23
	Fruits	0.00	0.10	0.01	0.04
	Nitrogen SD	0.00	0.95	0.10	0.38
	Carbon SD	1.17	3.88	2.29	2.45
