# Supplementary material for: Diet overlap and spatial segregation between two neotropical marsupials revealed by multiple analytical approaches
Source: PLoS One. 2017 Jul 12;12(7):e0181188. doi: 10.1371/journal.pone.0181188 (PMC5507539; doi:10.1371/journal.pone.0181188)
Supplement: S4 Table — This is the S4 Table legend. (PDF) [file pone.0181188.s004.pdf]

Table S4. Nestedness (NODF) and Modularity (Q) for *Didelphis aurita* and *Metachirus nudicaudatus* individual-resource networks.

	**Observed**	CI 95%	
		Lower	Upper
**NODF**			
raw	51.09	50.00	54.61
season	51.10	49.88	54.59
spring	65.06	61.30	68.85
summer	63.99	60.37	65.78
autumn	42.68	31.97	46.26
winter	47.85	42.07	48.57
**Q**			
raw	0.30	0.28	0.30
season	0.30	0.27	0.30
spring	0.22	0.21	0.25
summer	0.29	0.27	0.31
autumn	0.33	0.30	0.37

Raw: network analysis without grouping recaptures; *season*: network analysis grouping recaptures within seasons. *Spring*, *Summer*, *Autumn* and *Winter:* are network analysis for the respective season.
